# Human Laryngeal Mucus from the Vocal Folds: Rheological Characterization by Particle Tracking Microrheology and Oscillatory Shear Rheology

**DOI:** 10.3390/app11073011

**Published:** 2021-03-27

**Authors:** Gregor Peters, Olaf Wendler, David Böhringer, Antoniu-Oreste Gostian, Sarina K. Müller, Herbert Canziani, Nicolas Hesse, Marion Semmler, David A. Berry, Stefan Kniesburges, Wolfgang Peukert, Michael Döllinger

**Affiliations:** 1 Department of Otorhinolaryngology, Div. of Phoniatrics and Pediatric Audiology, University Hospital Erlangen, Friedrich-Alexander-University Erlangen-Nürnberg, 91054 Erlangen, Germany;; 2 Biophysics Group, Department of Physics, Friedrich-Alexander-University Erlangen-Nürnberg, 91052 Erlangen, Germany;; 3 Department of Otorhinolaryngology, University Hospital Erlangen, Friedrich-Alexander-University Erlangen-Nürnberg, 91054 Erlangen, Germany;; 4 Department of Chemical and Biological Engineering, Chair of Particle Technology, Friedrich-Alexander-University Erlangen-Nürnberg, 91058 Erlangen, Germany;; 5 Department of Head and Neck Surgery, David Geffen School of Medicine at University of California Los Angeles, Los Angeles, CA 90024, USA;

**Keywords:** human laryngeal mucus, viscoelasticity, particle tracking microrheology, oscillatory shear rheology, vocal folds

## Abstract

Mucus consistency affects voice physiology and is connected to voice disorders. Nevertheless, the rheological characteristics of human laryngeal mucus from the vocal folds remain unknown. Knowledge about mucus viscoelasticity enables fabrication of artificial mucus with natural properties, more realistic ex-vivo experiments and promotes a better understanding and improved treatment of dysphonia with regard to mucus consistency. We studied human laryngeal mucus samples from the vocal folds with two complementary approaches: 19 samples were successfully applied to particle tracking microrheology (PTM) and five additional samples to oscillatory shear rheology (OSR). Mucus was collected by experienced laryngologists from patients together with demographic data. The analysis of the viscoelasticity revealed diversity among the investigated mucus samples according to their rigidity (absolute G′ and G″). Moreover some samples revealed throughout solid-like character (G′ > G″), whereas some underwent a change from solid-like to liquid-like (G′ < G″). This led to a subdivision into three groups. We assume that the reason for the differences is a variation in the hydration level of the mucus, which affects the mucin concentration and network formation factors of the mucin mesh. The demographic data could not be correlated to the differences, except for the smoking behavior. Mucus of predominant liquid-like character was associated with current smokers.

## 1. Introduction

A healthy voice and speech are important for socializing and numerous professions. The social and economic disadvantages associated with severe voice and speech disorders are even more pronounced than for other communication disabilities like hearing malfunctions [1]. To overcome these drawbacks, appropriate treatment of voice and speech disorders is necessary, which requires a comprehensive understanding of voice and speech production.

In general, voice and speech production is an interplay of aerodynamics, mechanical dynamics and acoustics. The fluid-structure-acoustic interaction of the airflow from the lungs with the deformable tissue of the vocal folds creates the basic tone of voice in the larynx [2]. Supraglottal structures modulate it to the audible sound of the voice, known as speech [3].

Due to the complex nature of voice production, the reasons for voice disorders are numerous. Functional dysphonia, a preserved but disordered phonation, that is not directly caused by structural or neurologic pathologies, is a frequently observed voice disorder clinically. Aperiodic oscillations of the vocal folds or left-right asymmetries that affect glottal closure express it often. In that context, it was found that vocal fold hydration has an impact [4].

A mucus layer hydrates and lubricates the vocal folds, ensuring an accurate oscillation. In general, mucus covers the inner lining of organs, the epithelium, and serves as a protective barrier against pathogens, maintains the hydration of air-exposed organs, or ensures material transport [5]. Ex-vivo studies confirmed the impact of artificial mucus on the vibrational characteristics of the vocal folds, in relation to different viscoelastic properties [6,7]. Clinical studies reported mucus of varying thickness for persons with and without voice disorders [8,9]. Specific diseases like active laryngeal tuberculosis [10] or cystic fibrosis [11] reported voice disorders as effects of changed mucus consistency or mucus accumulation on the vocal folds. Vice versa, dehydration of the vocal folds was also found to affect oscillation characteristics [12].

The viscoelasticity of mucus depends on its composition [13]. For several organs, it was already determined. Human respiratory mucus was found by several studies to reveal gel characteristics [14–16]. Some studies investigated animal mucus that also revealed gel characteristics [16,17].

The mucins are the main component of mucus, that determine its viscoelasticity [18]. Mucins are glycoproteins that are able to build cross-linked complex hydrogels [16]. It is known that the polymer network of mucins is caused by hydrogen and disulfide bonds plus physical entanglements [19]. A study reported that low mucin concentrations of 1.5% leads to liquid-like mucus whereas a concentration of 5% results in solid-like mucus [20]. Besides the mucin concentration, the concentration of salts and surfactants and the pH affect the network building properties of mucins and thus mucus viscoelasticity [18,21] pulmonary diseases like cystic fibrosis, chronic obstructive pulmonary disorder and asthma affect the mucin content [15,22,23]. Nicotine was also found to affect the thickness of mucus gels [24]. In general, mucus consists of 1–5% of mucins, 90–95% water, 1% electrolytes, 1–2% lipids, other proteins, DNA, cells and cellular debris [5,25]. Similar mucin content at different locations in the human body results in similar rheological behavior, although the mucin glycoproteins themselves differ [22].

Detailed information about the viscoelasticity of mucus properties is rare. Limited amounts of available mucus collected from each patient restrict the use of bulk rheology. Particle tracking microrheology (PTM) is nowadays a commonly used technique to overcome the limitation of small sample amounts and determine rheological properties [26]. The viscoelastic properties determined by PTM depend on the diameter of the microspheres used in the experiments and their diffusion. Bulk rheology can be determined if the diameter of the microsphere exceeds the mesh size of the gel structure [22]. Particle tracking can also give information about the pore size of a gel network by using microspheres of different diameters. In the mucin network, microspheres of 500 nm diameter were entrapped, whereas smaller particles diffused freely through porcine respiratory mucus and human airway mucus [16,17]. Coating of the microspheres with polyethylenglycol was found to be advantageous to minimize mucoadhesion [14]. Lai et al. reviewed several studies of PTM of mucus, revealing mucus as a viscoelastic gel with low-viscosity fluid, between the entangled mucins [22].

The rheology of human laryngeal mucus has not been investigated yet, to the best of our knowledge. A detailed analysis will enable the design of an artificial mucus with natural viscoelastic properties. This will shed light on investigations regarding the impact of mucus on the oscillatory behavior of the vocal folds, phonation and the treatment of voice disorders. Our hypotheses for this study are:

1. Human laryngeal mucus from the vocal folds is viscoelastic and reveals gel characteristics, as mucus of another origin.
2. Variations of the viscoelasticity of the investigated mucus samples can be correlated with the demographic data.
3. Bulk rheology of human laryngeal mucus can be assessed by PTM with microspheres of appropriate diameter. The measurement results gained by PTM are comparable with OSR, the second applied measurement technique.

## 2. Materials and Methods

### 2.1. Mucus Samples

Mucus samples were collected from patients with a prerequisite age between 18 and 80 years. Patients with untreated metabolic diseases were excluded from the study. The samples were suctioned with a newly invented method consisting of a combination of a long rigid suction and a bronchio-alveolary lavage collection container during general anesthesia. A Kleinsasser tube was positioned in order to ensure maximal exposure of the larynx. The two surgeons were using a standardized scheme to warrant suctioning the two vocal folds in the same fashion for each patient. The good laryngeal exposure and the small tip of the suction ensured a specific sampling of vocal fold mucus only. The suctioning into the bronchio-alveolary lavage collection container guaranteed that the rheologic properties of the mucus were not compromised. The amount of each sample varied between 10 *μ*L and 1.8 mL.

Smears were taken and stained with hematoxylin and eosin before the samples were stored at −20 °C until measurements were performed. This enabled insight into the cell content and composition of the samples.

The patients suffered from different diseases of the upper respiratory tract. They were classified into two groups according to the presence of laryngeal pathologies: healthy larynx and pathologic larynx. Patients denoted with a pathologic larynx suffered from a pathology within the larynx, mostly a carcinoma.

Demographic data was collected from the patients, including gender, age and smoking behavior (cigarettes), to relate it to the viscoelasticity.

The study was approved by the local ethics commission (reference number: 425_18B). All patients gave their written consent to participate in the study.

### 2.2. Particle Tracking Microrheology

**Figure 1.**
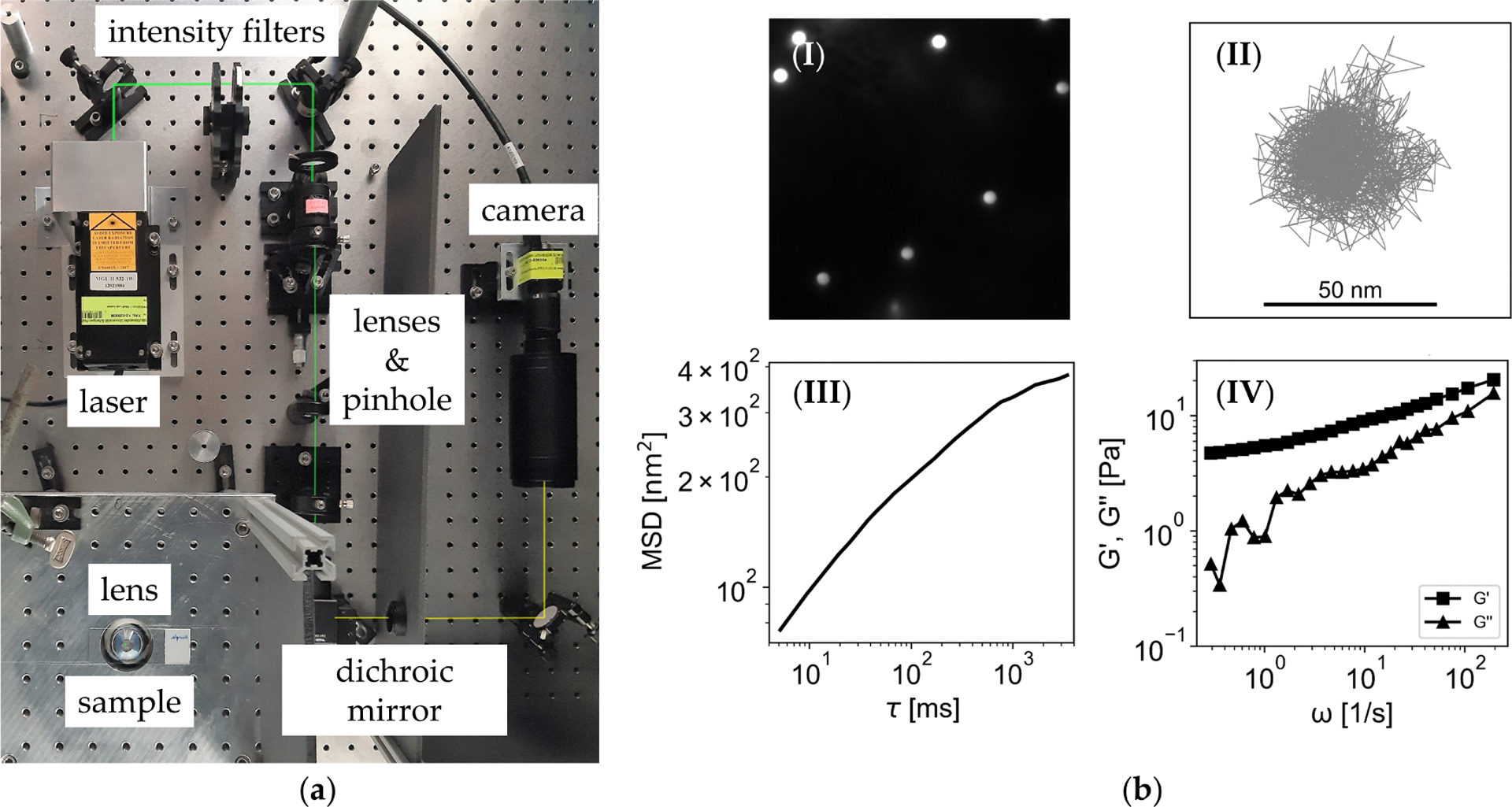
Particle tracking microrheology. (**a**) Measurement setup: A laser beam excited the fluorescent beads in the sample, which was placed on a lens with 100x magnification. A camera tracked the movement of the fluorescent microspheres. (**b**) Evaluation of the measurement data. (I) Fluorescent microspheres in the sample. (II) Trajectory of a microspheres’ movement. (III) Mean square displacement (MSD) of a microsphere over lag-times (*τ*). (IV) Viscoelastic properties, storage (G′) and loss modulus (G″) of the micorspheres surrounding medium, calculated of the MSD.

The measurement setup for passive PTM, see Figure 1a, was developed by the Biophysics Group, Department of Physics, FAU Erlangen. FluoSpheres^™^ (Thermo Fisher Scientific, Waltham, MA, USA), carboxylate-modified microspheres, orange fluorescent (540 nm/560 nm) with a diameter of 1.0 *μ*m were used for the measurements. A microsphere diameter of 1 *μ*m was estimated to be immobilized by the mucin mesh [17,22] to get bulk-rheological properties of the mucus. In order to prevent the microspheres from adhesion to the mucus ingredients, they were coated with polyethylenglycol (Mn 3000, Sigma-Aldrich, St. Louis, MO, USA), according to a procedure proposed by Suh et al. [27]. Of the 1% microsphere solution, 1–3 *μ*L were mixed with 10–30 *μ*L of mucus. Variations were caused due to the unsuitability of mucus for pipetting. The mucus-microsphere mixture was sealed between a glass slide and coverslip (gap width: 0.25 mm) by a Gene Frame (Thermo Fisher Scientific). The cover slip was placed onto a CFI Plan Apochromat microscopy objective lens (Nikon, Minato, Japan) with 100x magnification. A 1 W laser (532 nm) was used to excite the fluorescent microspheres. Filters, lenses and a pinhole controlled the intensity and focused the laser beam. Both laser beam and fluorescence passed a dichroic mirror. A Guppy Pro F-031B camera (Allied Vision Technologies GmbH, Stadtroda, Germany) was used to record the microsphere movements. The frame rate was 200 frames/s and 2 × 2 binning was used. The whole setup was placed on an active optical table to reduce vibrations.

The evaluation process of a microsphere is depicted in Figure 1b. The calculation of the viscoelastic properties of a microsphere surrounding the medium is based on the work of Mason et al. [28]. Single fluorescent beads (I) were tracked and the trajectory (II), was evaluated by an in-house software implemented in Python. Beads in close proximity to the surface were neglected due to possible adhesion to the cover slip. Linear trends of the microspheres movements due to thermal drifts were removed from the data. The mean square displacement (MSD, $\Delta {\bar{r}}^{2}(\tau )$) was calculated for each microsphere (III). The lag-times *τ* are defined periods of time over which the microspheres movement were evaluated. The MSD is approximated at each lag-time *τ*_0_ by a power-law function [26]

(1) $$\Delta {\bar{r}}^{2}(\tau )\approx \Delta {\bar{r}}^{2}(\tau ){\left(\frac{\tau }{\tau_{0}}\right)}^{\alpha \left(\tau_{0}\right)}$$

with *α* as the logarithmic slope of the MSD which equals the diffusive exponent.

(2) $$\alpha \left(\tau_{0}\right)={\frac{d\left(\text{ln}\Delta {\bar{r}}^{2}(\tau )\right)}{d(\text{ln}(\tau ))}|}_{\tau_{0}}$$

A microsphere’s movement is subdiffusive if 0 < *α* < 1, indicating a viscoelastic medium. In purely viscous fluids, *α* = 1 and in purely elastic solids, *α* = 0. Finally, the viscoelastic properties (IV) were determined by the Fourier transform of the MSD and the generalized Stokes–Einstein equation. In two-dimensional PTM, the complex shear modulus and subsequently the storage (G′) and loss modulus (G″) are calculated as follows [26]:

(3) $$\left|G^{*}\left(\omega_{0}\right)\right|={\frac{dk_{B}T}{3\pi R\text{ln}\Delta {\bar{r}}^{2}(\tau )\Gamma \left[\alpha \left(\tau_{0}\right)+1\right]}|}_{\tau_{0}=1/\omega_{0}}$$

(4) $$G^{\prime }(\omega )=\left|G^{*}(\omega )\right|\text{cos}(\pi \alpha (\omega )/2)$$

(5) $$G^{\prime \prime }(\omega )=\left|G^{*}(\omega )\right|\text{sin}(\pi \alpha (\omega )/2)$$

Each mucus sample was subdivided into three sub-samples. For each of the three sub-samples, 100 microspheres were tracked. This led to a total of 300 tracked microspheres in each mucus sample. The median was applied to the MSD of all 300 microspheres, followed by the calculation of *α* and the viscoelastic properties. The curves were smoothed by a moving average filter (width: 5 datapoints). The measurements were executed at room temperature (25 °C).

### 2.3. Oscillatory Shear Rheology

Measurements of OSR were performed using a TA Discovery Hybrid-Rheometer 2 with electrically heated plates (TA Instruments, New Castle, DE, USA). Parallel plates of a diameter of 25 mm were used and the measurement temperature was set to 25 °C. A water-soaked pipe cleaner mounted in the temperature hub created a damped atmosphere around the measuring system in order to avoid drying of the mucus. A gap of 0.5 mm was set with a short relaxation for the sample. The sample characterization was performed using small amplitude oscillatory shear (SAOS) via an amplitude and frequency sweep. For each mucus sample, an amplitude sweep was performed to determine the linear viscoelastic range. The measurement was aborted at a deformation of 10% to not irreversibly damage the structure of the mucus sample. Successively, a frequency sweep was performed within the linear viscoelastic range and frequencies of 0.01 Hz–100 Hz (0.06 rad/s–628 rad/s). The quality of the measurement data was assessed by Lissajous diagrams.

### 2.4. Statistical Analysis

Group values were not normally distributed. For multiple group comparisons, the Kruskal–Wallis test was used and a significance level of *p* = 0.05 was chosen. The Dunn-Bonferroni correction was applied for post-hoc tests (Mann-Whitney-U). The correction factor was selected according to the number of tests, i.e., n = 3 results in a significant *p*-value of 0.017.

## 3. Results and Discussion

### 3.1. Mucus Samples

Several mucus samples appeared to be heterogeneous. Staining of the mucus smears with hematoxylin and eosin revealed insight into the cell content. Squamous cells confirmed their origin from the vocal folds, but various extents of blood cells were also found. Purification of the mucus with common techniques like centrifugation or filtering methods could not be achieved without the destruction of the mucus gel structure. Thus, impure and heterogeneous samples were excluded from further analysis. This led to a total number of 24 samples that were applied to the rheological analysis.

Five samples with a volume ≥300 *μ*L suited OSR: Two samples were from female donors, three from males, one of age <50, two of age 50–59, one of age 60–69 and one of age >70. The larynges of four patients were healthy and one larynx was pathologic. Two of the patients were smokers, two former smokers and one non-smoker.

The samples investigated by PTM and OSR were from different patients due to the limited size of mucus samples. We applied 19 mucus samples to PTM. The demographic data of the patients belonging to the mucus samples are given in Table 1.

### 3.2. Viscoelastic Properties

#### 3.2.1. Particle Tracking Microrheology

**Figure 2.**
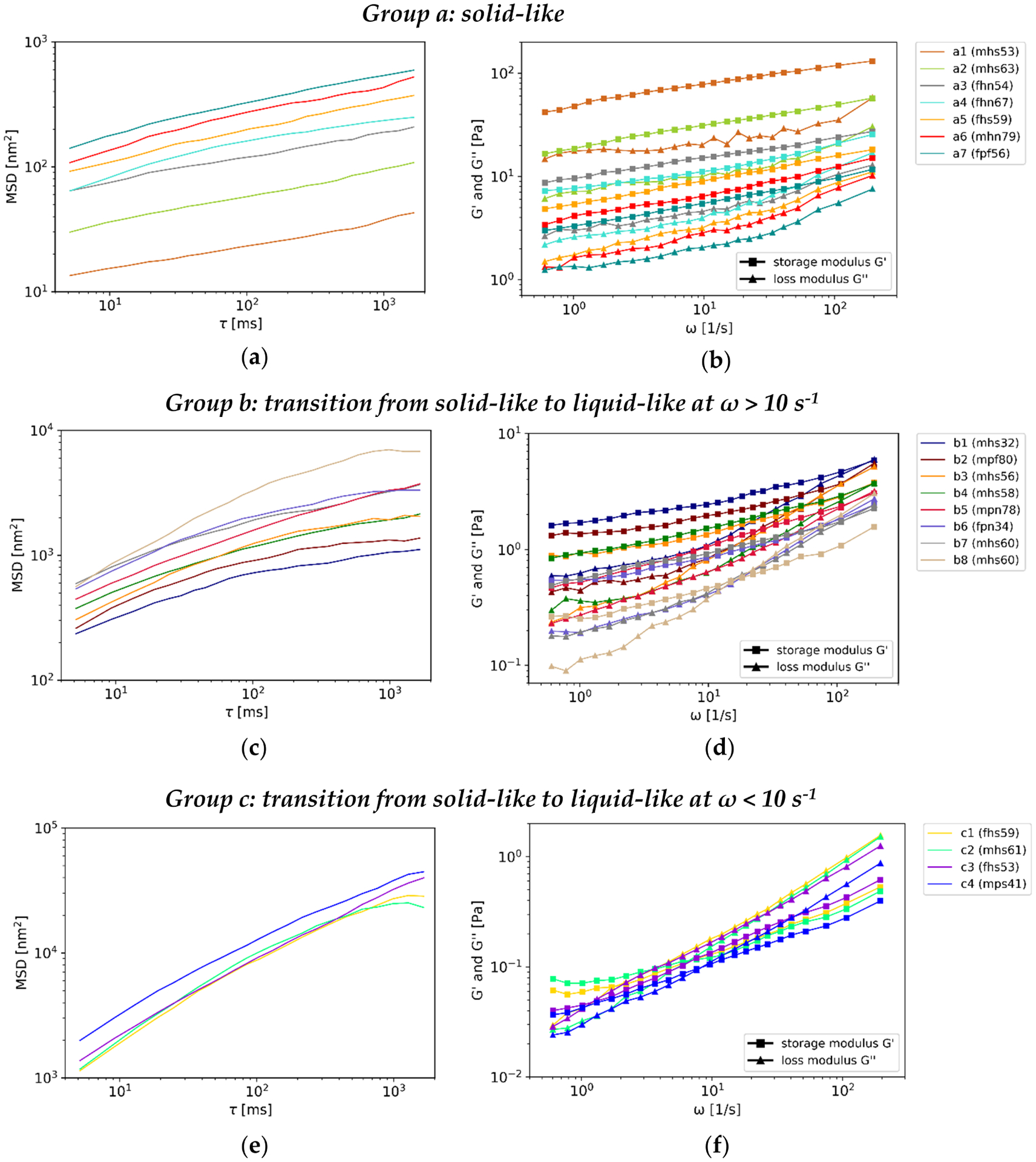
MSDs and viscoelastic properties of mucus samples, subdivided into three groups. (**a**) MSD group a; (**b**) viscoelasticity group a; (**c**) MSD group b; (**d**) viscoelasticity group b; (**e**) MSD group c; (**f**) viscoelasticity group c. Group a mucus samples revealed the lowest MSDs, the highest loss and storage moduli and throughout gel character. MSDs increased from group a over b to c. Hence the storage and loss moduli decrease. Group b and c mucus samples revealed a crossover of the moduli. The abbreviations in the brackets give information about the patients’ demographic data belonging to the mucus samples: 1. digit: gender (f : female, m: male); 2. digit: larynx status (h: healthy, p: pathologic); 3. digit: smoking behavior (s: smoker, f: former smoker, n: non-smokers); 4/5. digit: age.

**Figure 3.**
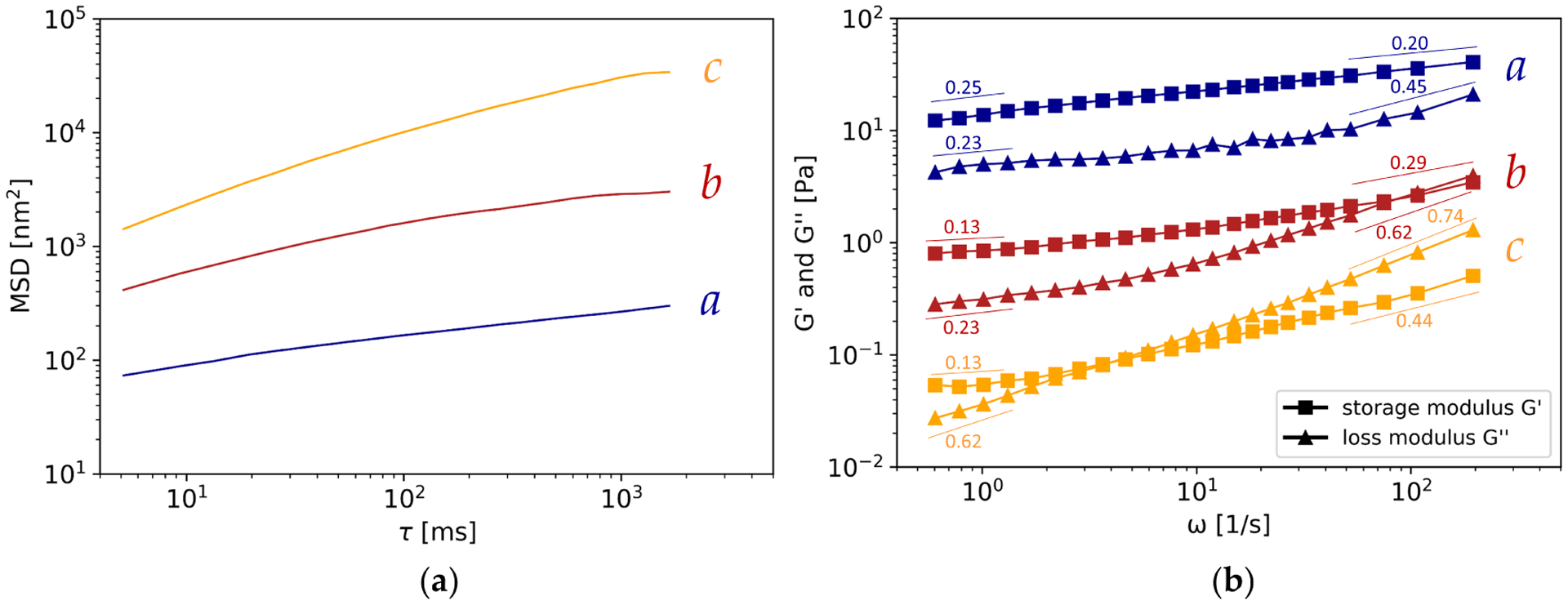
Mean of the MSDs and viscoelastic properties with slopes of the three groups. blue: group a; red: group b; yellow: group c. (**a**) Averaged MSDs; (**b**) Averaged viscoelastic properties. A crossover can be seen within groups b and c, occurring at a lower frequency for group c. The values given near the evaluation limits for each curve reveal the mean slope over the four measurement points near the evaluation limits.

The MSDs and absolute viscoelastic properties, G′ and G″, which are referred to the rigidity of the mucus samples, varied over four orders of magnitude and report the diversity of human laryngeal mucus. Based on the absolute MSDs, the according diffusive exponents and corresponding viscoelastic characteristics, especially a crossover of the storage and loss modulus (G′, G″), the results led to a subdivision of the mucus samples into three groups: a, b and c, see Figure 2. The mean MSDs and the mean storage and loss moduli, including their slopes over the four data points near the evaluation limits, were calculated for each group, see Figure 3. The according mean absolute MSDs and viscoelastic properties at the evaluation limits (i.e., *τ* = 5.14 ms, *τ* = 1662 ms and *ω* = 0.6 s^−1^, *ω* = 195 s^−1^) are given in Table 2.

Group a mucus samples were characterized by the lowest MSDs and highest storage and loss moduli compared to group b and group c mucus samples, see Figure 2. This can be seen more distinct in the direct comparison of the mean curves of the groups, see Figure 3. The according characteristic parameters of the MSD and viscoleasticity at the evaluation limits, see Table 2, underline the visualization. Group a mucus samples revealed the lowest diffusive exponents *α*. Information about the rigidity of gels at rest, the long-term behavior, is given by G′ and G″ at the lowest evaluated frequency. These are higher for group a than for the other two groups: At (*ω* = 0.6 s^−1^), G′ = 12.28 Pa and G″ = 4.19 Pa. Nevertheless, the mean G′ and G″ revealed high standard deviations, which are caused by varying absolute moduli of the samples within the group, see Figure 2. In this context, the extreme cases of group a were mucus samples a1 and a7. The extreme cases caused a range of G′ from 3.0 Pa to 42.07 Pa and G″ from 1.24 Pa to 14.76 Pa at the lower evaluation limit (*ω* = 0.6 s^−1^) and G′ from 11.71 Pa to 131.30 Pa and G″ from 7.54 Pa to 57.72 Pa at the higher evaluation limit (*ω* = 194 s^−1^), see Appendix A, Table A1. The loss factor tan*δ* = G″/G′, which describes the relationship of the moduli, was throughout smaller than 1 for group a mucus samples. G′ and G″ increased with increasing frequency. The slope of the mean G′, given in Figure 3, revealed a decrease which resulted in a flattening of the curve, whereas the slope of G″ increased over frequency.

Group a mucus samples revealed classic gel character. The elastic modulus G′ was higher than the storage modulus G″ over the whole range of evaluated frequencies, which reveals solid-like, elastic-dominant character (G′ > G″). This is in accordance with bulk-rheology measurements of different kinds of mucus reported previously [14–17]. The absolute storage and loss modulus varied within the samples in group a. However, their range cover rigidity of native human airway mucus [14–16] and native intestinal mucus of pigs [16] found before.

Group b mucus samples revealed higher MSDs than group a, lower MSDs than group c mucus samples and vice versa lower storage and loss moduli than group a and higher than group c, see Figures 2 and 3. Additionally, a crossover at frequencies *ω* > 10 s^−1^ can be seen. The crossover led to a change from tan*δ* < 1 at the lower evaluation limit to tan*δ* > 1 at the higher limit, see Table 2. The mean diffusive exponent *α* lies between group a and group c at the lowest lag-time *τ* = 5.14 ms, but is almost equal to group a and lower than group c at the highest lag-time. The rigidity at rest (G′ and G″ at *ω* = 0.6 s^−1^) lies in between group a and c: G′ = 0.80 Pa, G″ = 0.28 Pa. Extreme cases in this group could be found according to the absolute moduli and the crossover frequency. The highest absolute moduli were found for mucus sample b1 and the lowest moduli were found for mucus sample b7 and b8, depending on the frequency. This leads to a range of G′ from 0.26 Pa to 1.62 Pa and G″ from 0.1 Pa to 0.59 Pa at the lower evaluation limit (*ω* = 0.6 s^−1^) and G′ from 1.57 Pa to 5.86 Pa and G″ from 2.43 Pa to 5.97 Pa at the higher evaluation limit (*ω* = 194 s^−1^), see Appendix A, Table A1. The crossover at the lowest frequency was found for mucus sample b8, followed by sample b3, which showed slightly different characteristics within the group. For these two samples, the crossover happened between frequencies of *ω* = 10 s^−1^ and *ω* = 100 s^−1^. For the other samples, the crossover happened at frequencies *ω* > 100 s^−1^. The slopes of the mean curves of G′ and G″, given in Figure 3, reveal an increase over frequency. The increase is more distinct for the slope of G″ than G′ which leads to the crossover of the moduli.

Group b mucus samples revealed lower rigidity as group a due to lower absolute G′ and G″. For the mean frequency range, solid-like behavior can be found for most of the samples, which is typical for gels and in accordance to group a mucus samples. Neverthless, at higher frequencies, a convergence and finally crossover of G′ and G″, a transition from a solid-like character to a liquid-like, viscous-dominant character (G″ > G′), happened. We assume that this may be linked to the mucin network. The rheological properties determined by PTM are based on the microspheres thermal diffusion and are directly affected by the microstructure of the sample. High frequencies are related to the short lag-times over which the microspheres movement was evaluated. Thus, short lag-times are connected to the local diffusion of the microspheres. The liquid-like character at high frequencies, is an indication for a less hindered diffusion of the microspheres over short times, compared to frequencies at which the elastic component (G′) dominates. We assume that the crossover happens due to a looser mucin network and differences in the composition of the mucus samples of group b in comparison to group a. Several factors as pH, surfactant-concentration or salt concentration were reported to affect the gel building properties of mucins [21]. Moreover, the mucin content itself is the main component that affects the viscoelasticity of mucus [18,20]. In the context of the mucus ingredients, the hydration level of mucus can be responsible for diluted or concentrated mucus. We assume that this is the reason for the observed differences.

Group c mucus samples revealed the highest MSDs and lowest viscoelastic moduli compared to the other two groups, see Figures 2 and 3. A crossover of the moduli could be observed at frequencies *ω* < 10 s^−1^, frequencies lower than for group b mucus samples. As for group b, this leads to a change of tan*δ*, see Table 2. The highest diffusive exponents, at both evaluation limits, were found for group c. The absolute moduli of the samples within the group did not vary much, which is represented by the small standard deviation. A range of G′ from 0.037 Pa to 0.40 Pa and G″ from 0.02 Pa to 0.03 Pa at the lower evaluation limit (*ω* = 0.6 s^−1^) and G′ from 0.40 Pa to 0.62 Pa and G″ from 0.87 Pa to 1.56 Pa at the higher evaluation limit (*ω* = 194 s^−1^) were found, see Appendix A, Table A1. The crossover frequency was the lowest for mucus sample c4 and the highest for mucus sample c2. The slopes of the mean curves of G′ and G″ increase with frequency, see Figure 3. The mean storage and loss modulus at the lower evaluation limit were smaller than for groups a and b.

Group c mucus samples revealed the lowest absolute G′ and G″ compared to group a, and group b. The liquid-like character dominates. However, at low frequencies, solid-like character was found. The crossover of G′ and G″ happened at lower frequencies than for group b. This may be due to an even looser mucin network, compared to group b mucus samples.

A statistical analysis, based on characteristic parameters at the limits of evaluation (Table 2), was performed to check on the independence of the proposed groups. The results are given in Table 3. All mean parameters, except the diffusive exponent *α* at the higher evaluation limit (*τ* = 1662 ms) and tan*δ* at the lower evaluation limit (*ω* = 0.6 s^−1^), showed differences among the groups (bold). The same six differences were found for group a versus group b, group a vs. group c and group b vs. group c mucus samples. The analysis validated the classification.

In sum, the absolute MSDs and the diffusive exponents *α* increased from group a over group b to group c. The subdiffusive movement (*α* < 1, see Table 2) of the microspheres, revealed the viscoelastic character of the human laryngeal mucus samples. Vice versa, the storage (G′) and loss moduli (G″) decreased from group a over group b to group c. For all mucus samples of all groups, tan*δ* is smaller than 1 at the lower evaluation limit. One can conclude that the rigidity and the mucin-network properties of the investigated samples differ. We assume that the differences were caused by variations of the hydration level of the mucus samples

The trends of G′ and G″ including the crossover, can be interpreted in the context of entangled polymer solutions. Entangled polymer solutions show a glassy (G′ > G″), a rubbery (G′ > G″) and a flow regime (G′ < G″) with increasing temperature or decreasing frequency. Additionally, a transition regime is found between the rubbery and glassy region, which is characterized by (G′ < G″) [29]. The solid-like character in our findings can be related to the rubbery regime and the liquid-like character to the transition regime.

The convergence (group a) and the crossover (group b, group c) of G′ and G″ may lead to the assumption that mucus has probable viscous characteristics at high shear, serving as a lubricant for the vocal folds during oscillation but exhibiting viscoelastic solid characteristics at rest. Although this would presuppose a fast regeneration of the gel after destruction. A rapid reformation of the gel of pig gastric mucus after the destruction was already found [30]. However, further investigations would be necessary to substantiate these assumptions due to the high forces acting on mucus during oscillation of the vocal folds.

**Figure 4.**
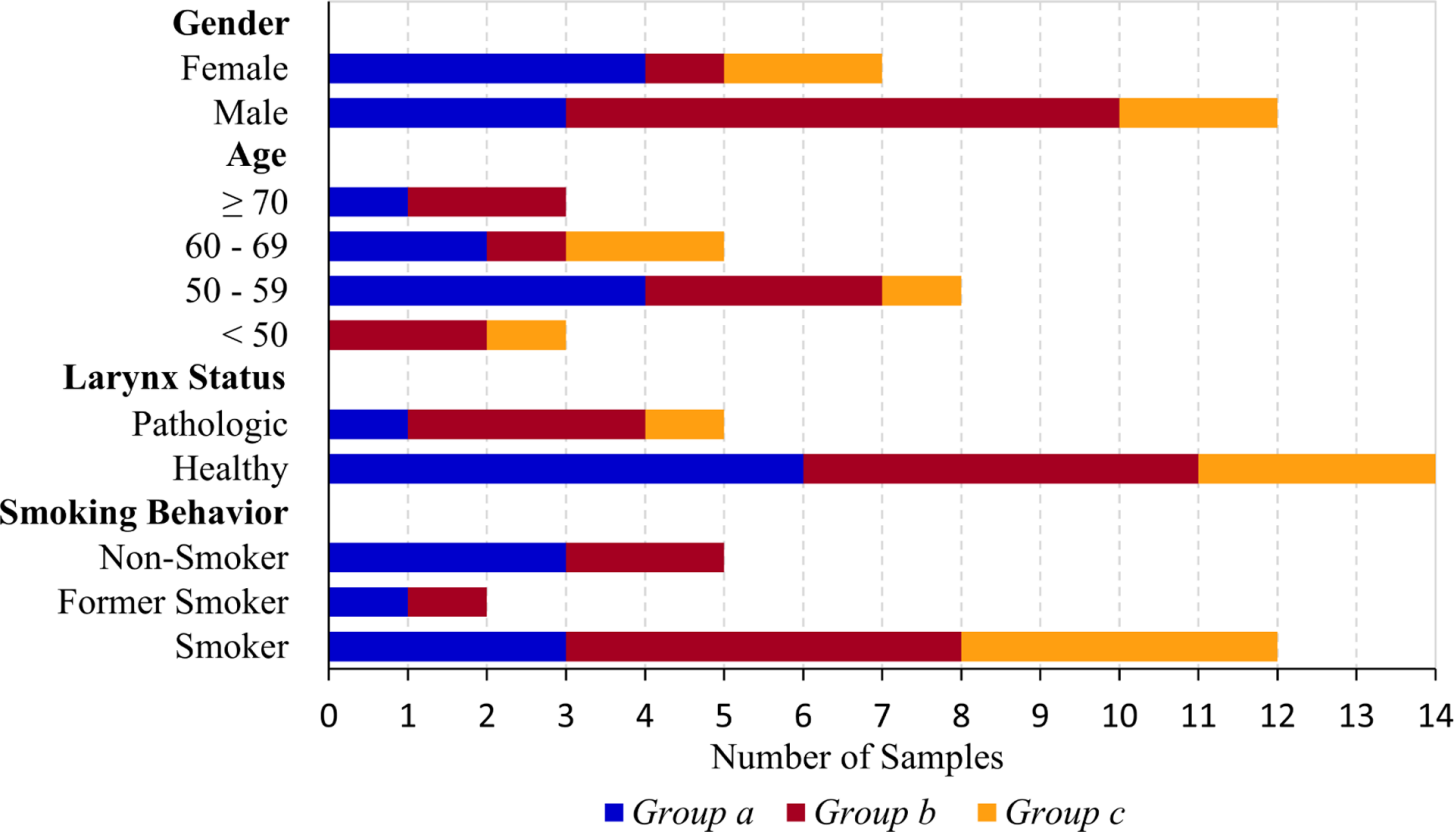
Relationship of the demographic data of the patients to the mucus groups. All groups were found for different gender and larynx status. Whereas group a and group b mucus samples were found for all ages and all smoking behaviors, group c mucus samples were only found for smokers and patients <70 years.

The relationship of the demographic data of the patients belonging to the mucus samples and groups is captured in Figure 4. Group a, group b and group c mucus samples were found for both gender and both larynx status. For patients older than 70 years, no group c mucus samples were found, but group a and group b mucus samples. For the other age ranges, all three groups were present. Group c mucus samples were only found for current smokers, not for non-smokers or former smokers. Group a and group b mucus samples were related to all smoking behaviors.

This leads to the assumption that there are no tendencies with respect to gender and laryngeal status. A firm correlation with respect to age cannot be firmly established due to the limited samples. It is remarkable that group c mucus was only found for current smokers. Nevertheless, this is not in accordance with a study done before, where nicotine was found to lead to higher mucus viscosity [24]. However, a relationship between smoking and the rigidity of laryngeal mucus from the vocal folds seems reasonable.

#### 3.2.2. Oscillatory Shear Rheology

**Figure 5.**
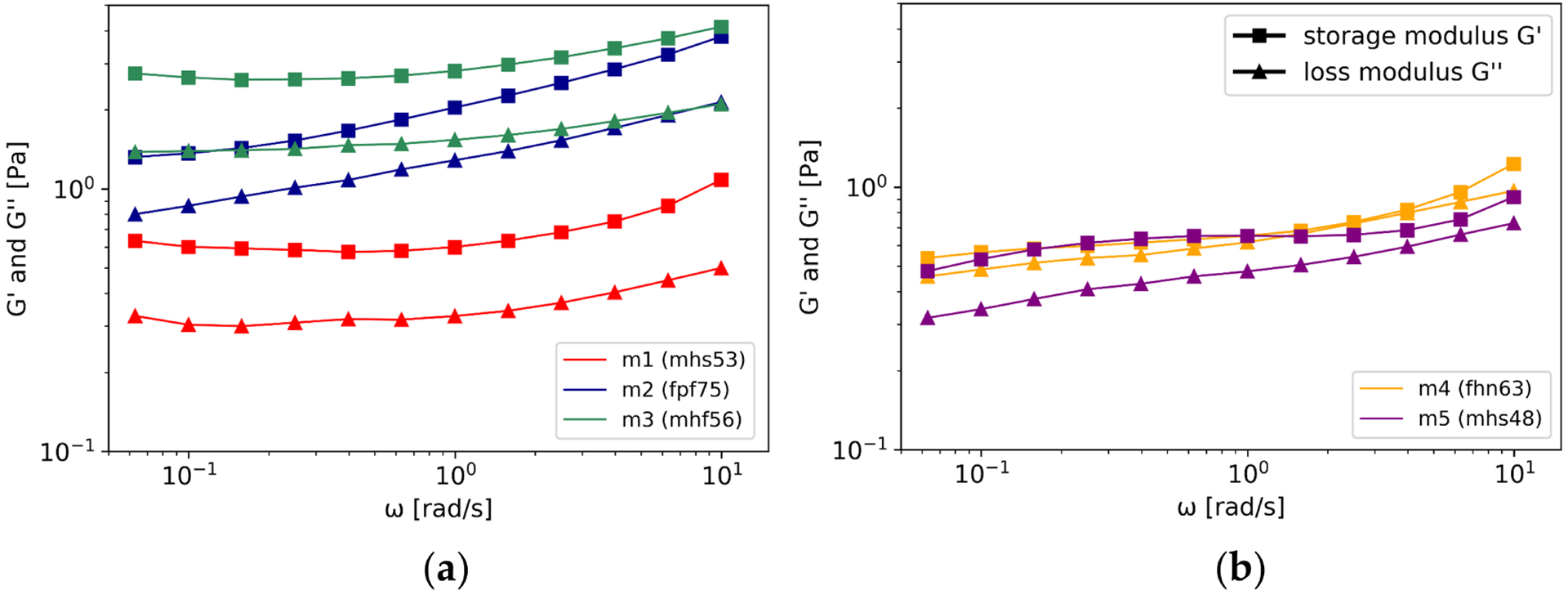
(**a**,**b**): Measurement results of oscillatory shear rheology (OSR). Four of the five investigated mucus samples revealed gel characteristics (m1, m2, m3, m5). One sample revealed close moduli (m4). The abbreviations in the brackets give detailed information about the demographic data of the patients belonging to the samples: 1. digit: gender (f : female, m: male); 1. digit: larynx status (h: healthy, p: pathologic); 3. digit: smoking behavior (s: smoker, f: former smoker, n: non-smokers); 4/5. digit: age.

The viscoelastic properties determined by OSR are depicted in Figure 5. The quality of the measurement data was assessed by Lissajous diagrams, which led to an exclusion of frequencies above 10 rad/s. The absolute storage and loss moduli of the mucus samples differed. At rest (*ω* 0.06 rad/s), all mucus samples showed higher storage than loss modulus, see Table 4. The highest storage and loss modulus was found for mucus sample m3, G′(0.06 rad/s) = 2.75 Pa and G″(0.06 rad/s) = 1.38 Pa. The lowest moduli were found for m5, G′(0.06 rad/s) = 0.48 Pa and G″(0.06 rad/s) = 0.32 Pa. Four of the five mucus samples consistently showed a higher storage than loss modulus (m1, m2, m3, m5). Sample m4 revealed a higher storage than loss modulus at low, but almost equal moduli at high frequencies. No subdivision was attempted due to limited samples (n = 5). The demographic data of the patients belonging to the samples are given in brackets in the legends.

#### 3.2.3. Comparison of Particle Tracking Microrheology and Oscillatory Shear Rheology

As mentioned before, microspheres of 1 *μ*m diameter were chosen to get information about bulk-rheological properties by PTM. This allowed a direct comparison to OSR.

All mucus samples measured by PTM and OSR revealed higher storage than loss modulus (G′ > G″) and tan*δ* < 1 at rest (the lowest evaluated frequencies). This characterizes the long-term behavior of human laryngeal mucus as a viscoelastic solid or gel. Nevertheless, the absolute G′ and G″ and tan*δ* differed, which indicates varying rigidity of the mucus samples.

**Figure 6.**
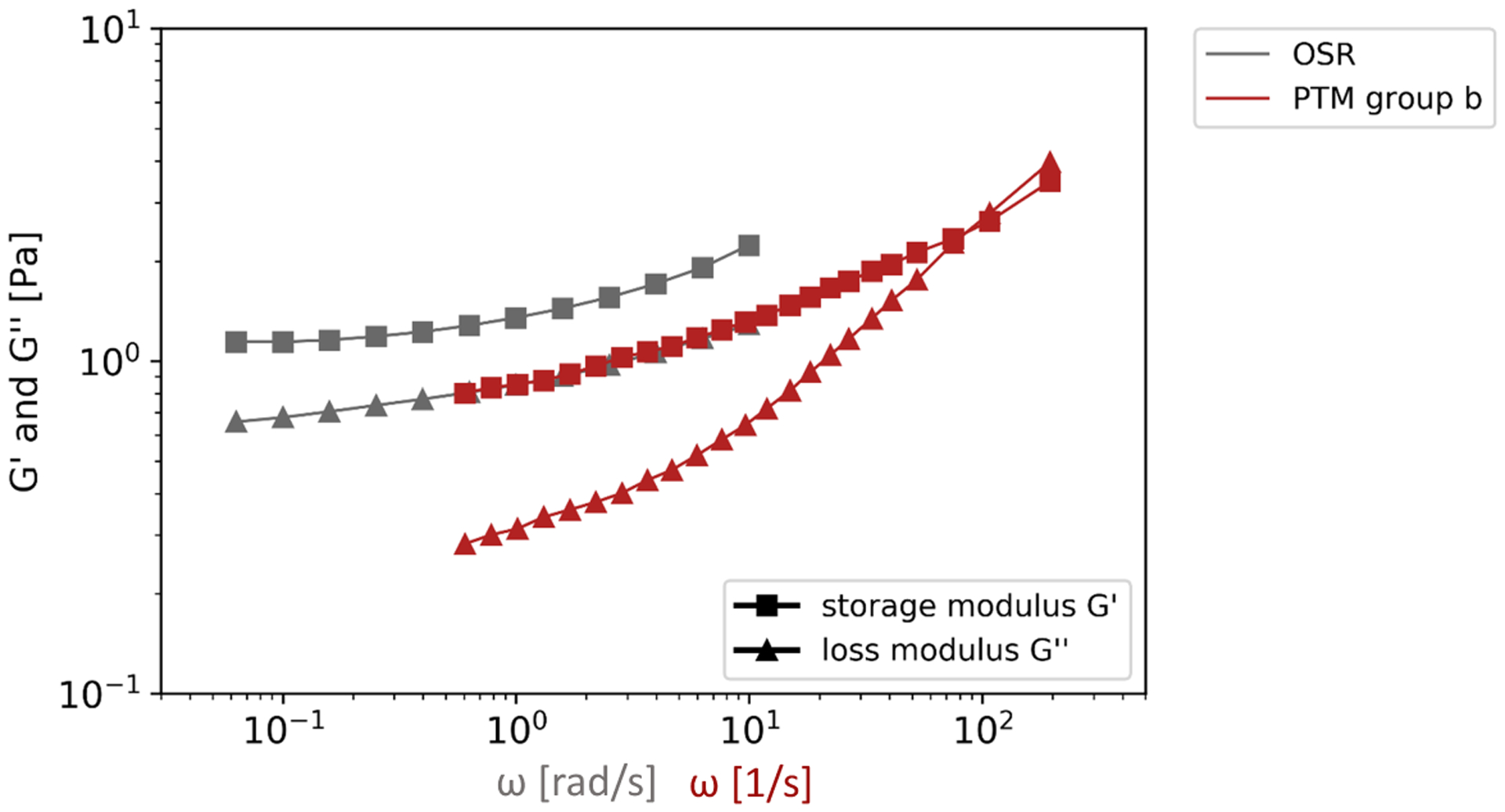
Comparison of the mean viscoelasticity of all OSR measurements and the mean of PTM measurements, group b.

The absolute G′ and G″ of mucus samples applied to OSR resembled most group b mucus samples, applied to PTM, see Figure 6. The absolute moduli were lower than for respiratory mucus measured by OSR before [14–16]. An agreement could be found for porcine airway mucus [17]. Neverthless, consistent gel character was found for four of the five mucus samples. This is in accordance with the previously reported viscoelasticity of human airway mucus.

## 4. Shortcomings

The number of available samples for the study was limited and the total number of suitable samples for the analysis was relatively small. Furthermore, the volume of aspirated mucus collected from each patient was not enough to apply both PTM and OSR measurements to the same mucus samples.

The mesh-size of the mucin network was not determined by microspheres of different diameter or cryo-scanning electron microscopy. Additionally, the water content of the samples was not determined by drying of the samples.

## 5. Conclusions

This study investigated the rheological properties of human laryngeal mucus from the vocal folds. It is the first time that mucus was aspirated directly from the vocal folds and characterized rheologically. Due to the limited sample volumes, particle-tracking microrheology (PTM) was applied and the viscoelastic characteristics of 19 mucus samples were evaluated. Oscillatory shear rheology (OSR) was applied and evaluated for five additional mucus samples. The results can be summarized as follows:

1. All investigated human laryngeal mucus samples presented as viscoelastic solids, at rest with varying rigidity, independent of the measuring method.
2. PTM led to a preliminary subdivision of the samples into three groups with different rigidity. Seven of the 19 investigated samples (group a) revealed consistent solid-like character, which is typical for gels. Their absolute storage and loss modulus were in the range of native human airway mucus investigated before [14–16]. For the other samples (group b, group c), a transition from solid-like to liquid-like character was found. The transition happened for group b samples at high frequencies, characterizing the samples predominantly as viscoelastic-solids. For group c mucus samples, the crossover was found at low frequencies, characterizing the samples predominantly as viscoelastic-liquids. We assume that this is caused by a looser mucin network, as a consequence of a varying hydration level of the samples. The hydration affects the concentration of the main gel-building component of mucus, the mucins [18,20], and according network-building factors as pH, salt and surfactant concentration [21].
3. A correspondence between the groups and the demographic data of the patients belonging to the samples was not found for gender, age and larynx status but was found regarding the smoking behavior. The mucus samples with the lowest rigidity (group c) were only assigned to current smokers.
4. OSR revealed consistent gel characteristics for four of the five investigated mucus samples. The rigidity of the gels varied. The absolute viscoelastic properties were in the range of PTM and could be assigned to group b mucus samples.

The findings in this study reveal varying viscoelastic characteristics of human laryngeal mucus from the vocal folds and create a basis for the design of artificial mucus with natural viscoelastic properties. It can be expected that the characteristic properties of laryngeal mucus are governed by scaling laws similar to these known in harvested bronchial epithelial mucus [20] or in living cells [31,32], which facilitates the creation of highly adaptable and well controllable substitutes. Although artificial mucus was already found to have an impact on the oscillatory behavior of the vocal folds [7], this study more firmly establishes a foundation for future ex-vivo larynx experiments with realistic viscoelastic conditions of mucus. This will permit the examination of the influence of mucus characteristics on laryngeal dynamics, and ultimately on clinical populations with functional dysphonia. Finally, the presented results may serve as a basis for artificial mucus fabrication for patients suffering from oral dehydration [33,34].

## Figures and Tables

**Table 1.** Demographic data of the patients belonging to the 19 mucus samples measured by particle tracking microrheology (PTM). Number of evaluated samples based on gender, age, larynx status and smoking behavior.

**Gender**	**Male**		**Female**	
	12		7	
**Age**	**<50**	**50–59**	**60–69**	**≥70**
	3	8	5	3
**Larynx Status**	**Healthy**		**Pathologic**	
	14		5	
**Smoking Behavior**	**Non-Smokers**	**Former Smokers**		**Smokers**
	5	2		12

**Table 2.** Characteristic mean parameters of the MSDs and viscoelasticity. MSD parameters: absolute MSD and diffusive exponent at the evaluation limits *τ* = 5.14 ms and *τ* = 1662 ms. Viscoelasticity parameters: Storage modulus G′, loss modulus G″ and tan*δ* at *ω* = 0.6 s^−1^ (at rest) and tan*δ* at *ω* = 195 s^−1^.

		Group a	Group b	Group c
**MSD Parameters**				
*τ* = 5.14 ms:	MSD [nm^2^]	73 ± 41	451 ± 128	1420 ± 341
	*α*	0.33 ± 0.04	0.57 ± 0.07	0.76 ± 0.04
*τ* = 1662 ms:	MSD [nm^2^]	300 ± 191	3489 ± 1709	34036 ± 8632
	*α*	0.21 ± 0.03	0.22 ± 0.04	0.32 ± 0.07
**Viscoelasticity Parameters**				
*ω* = 0.6 s^−1^ (at rest):	G′ [Pa]	12.28 ± 12.89	0.80 ± 0.43	0.05 ± 0.02
	G″ [Pa]	4.19 ± 4.60	0.28 ± 0.15	0.03 ± 0.00
*ω* = 195 s^−1^:	tan *δ*	0.34 ± 0.05	0.36 ± 0.06	0.55 ± 0.15
	tan *δ*	0.58 ± 0.09	1.21 ± 0.30	2.57 ± 0.46

**Table 3.** Statistical analysis of characteristic MSD and viscoelasticity parameters. Differences were found for all parameters except the diffusive exponent *α* at the higher and the tan*δ* at the lower evaluation limit and comparison of all groups.

		Post Hoc Tests (Mann-Whitney-U-Test; *p* < 0.017)			Kruskal Wallis (*p* < 0.05)
		*Group a vs. b*	*Group a vs. c*	*Group b vs. c*	
**MSD Parameters**					
*τ* = 5.14 ms:	MSD	**0.000**	**0.006**	**0.004**	**0.000**
	*α*	**0.000**	**0.006**	**0.004**	**0.000**
*τ* = 1662 ms:	MSD	**0.000**	**0.006**	**0.004**	**0.000**
	*α*	-	-	-	0.149
**Viscoelasticity Parameters**					
*ω* = 0.6 s^−1^ (at rest):	G′	**0.000**	**0.006**	**0.004**	**0.000**
	G″	**0.000**	**0.006**	**0.004**	**0.000**
*ω* = 195 s^−1^:	tan*δ*	-	-	-	0.149
	tan*δ*	**0.000**	**0.006**	**0.004**	**0.000**

**Table 4.** Storage (G′), loss (G″) moduli and tan*δ* of the mucus samples investigated by OSR at the evaluation limits of *ω* = 0.06 rad/s and *ω* = 10 rad/s.

	*ω* = 0.06 rad/s			*ω* = 10 rad/s		
Sample	G′ [Pa]	G″ [Pa]	tan*δ*	G′ [Pa]	G″ [Pa]	tan*δ*
**m1**	0.63	0.33	0.52	1.08	0.50	0.46
**m2**	1.32	0.80	0.61	3.80	2.14	0.56
**m3**	2.75	1.38	0.50	4.15	2.10	0.51
**m4**	0.54	0.46	0.85	1.23	0.97	0.79
**m5**	0.48	0.32	0.66	0.92	0.73	0.79

## Appendix A

**Table A1.** MSD and diffusive exponents *α* at the evaluation limits *τ* = 5.14 ms and *τ* = 1662 ms, G′, G″ and tan*δ* at *ω* = 0.6 s^−1^ and *ω* = 195 s^−1^ for all mucus samples. The superscripted digits give information about the demographic data of the patients, belonging to the mucus samples: 1. digit: gender (f : female, m: male); 2. digit: larynx status (h: healthy, p: pathologic); 3. digit: smoking behavior (s: smoker, f: former smoker, n: non-smokers); 4/5. digit: age.

	*τ* = 5.14 ms		*τ* = 1662 ms		*ω* = 0.6 s^−1^			*ω* = 195 s^−1^		
Sample	MSD [nm^2^]	*α*	MSD [nm^2^]	*α*	G′ [Pa]	G″ [Pa]	tan*δ*	G′ [Pa]	G″ [Pa]	tan*δ*
**a1**^*mhs*53^	13.48	0.26	42.85	0.21	42.07	14.76	0.35	131.30	57.72	0.44
**a2**^*mhs*63^	30.01	0.31	108.30	0.22	16.60	6.10	0.37	57.43	30.39	0.53
**a3**^*f hn*54^	64.30	0.28	208.42	0.19	8.70	2.64	0.30	27.28	12.90	0.47
**a4**^*f hn*67^	64.23	0.37	249.01	0.15	7.31	1.76	0.24	25.47	16.96	0.67
**a5**^*f hs*59^	92.15	0.35	372.90	0.19	4.86	1.50	0.31	18.16	11.07	0.61
**a6**^*mhn*79^	108.18	0.38	524.41	0.24	3.42	1.32	0.39	15.04	10.21	0.68
**a7**^*f p f*56^	140.96	0.36	594.19	0.25	3.00	1.24	0.41	11.71	7.54	0.64
**b1**^*mhs*32^	235.54	0.51	1112.81	0.22	1.62	0.59	0.37	5.86	5.97	1.02
**b2**^*mp f*80^	260.46	0.52	1372.63	0.20	1.32	0.43	0.33	5.16	5.53	1.07
**b3**^*mhs*56^	305.89	0.60	2049.39	0.17	0.89	0.24	0.27	3.77	5.17	1.37
**b4**^*mhs*58^	375.42	0.50	2141.22	0.22	0.84	0.30	0.35	3.70	3.73	1.01
**b5**^*mpn*78^	445.83	0.51	3732.58	0.29	0.47	0.23	0.49	3.07	3.18	1.04
**b6**^*f pn*34^	538.98	0.54	3321.64	0.22	0.54	0.20	0.36	2.43	2.73	1.12
**b7**^*mhs*60^	593.90	0.52	3675.53	0.22	0.49	0.18	0.37	2.26	2.43	1.07
**b8**^*mhs*60^	561.99	0.70	6782.61	0.23	0.26	0.10	0.37	1.57	3.04	1.94
**c1**^*f hs*59^	1141.26	0.79	28450.66	0.28	0.06	0.03	0.48	0.53	1.56	2.93
**c2**^*mhs*61^	1175.78	0.80	23114.57	0.21	0.08	0.03	0.35	0.49	1.52	3.11
**c3**^*f hs*53^	1373.96	0.71	39911.90	0.40	0.04	0.03	0.72	0.62	1.25	2.03
**c4**^*mps*41^	1991.76	0.73	44669.08	0.37	0.04	0.02	0.66	0.40	0.87	2.20
